# Improving vascular retention of indocyanine green for *in vivo* two-photon microscopy using liposomal encapsulation

**DOI:** 10.1117/1.JBO.30.9.096004

**Published:** 2025-09-23

**Authors:** Alankrit Tomar, Noah Stern, Tyrone Porter, Andrew K. Dunn

**Affiliations:** a The University of Texas at Austin, Department of Electrical and Computer Engineering, Austin, Texas, United States; b The University of Texas at Austin, Department of Biomedical Engineering, Austin, Texas, United States

**Keywords:** two-photon microscopy, indocyanine green, liposomal encapsulation

## Abstract

**Significance:**

Two-photon microscopy is widely used for *in vivo* imaging of vasculature in rodents and requires the labeling of blood plasma with fluorescent dyes such as indocyanine green (ICG). However, a major limitation of ICG is its rapid clearance from the body, which restricts its use in extended imaging sessions. We address and overcome that limitation, enabling longer *in vivo* imaging sessions.

**Aim:**

We aim to investigate the feasibility of using liposomal nanoparticles that, when used to encapsulate ICG, significantly increase the circulation time of the vascular label in the rodent body.

**Approach:**

We conducted *in vivo* imaging experiments with unencapsulated (free) ICG and liposomal ICG (L-ICG) and compared the retention of ICG in the vascular network over a duration of 75 min.

**Results:**

In comparison to a retention time of around 20 min for free ICG, we find that liposomal encapsulation improves the vascular retention time of the dye to at least 75 min. The improvement in retention time using the encapsulation technique was consistent across imaging experiments conducted in five mice.

**Conclusion:**

The rapid clearance of ICG from the rodent body can be overcome using liposomal encapsulation, making prolonged *in vivo* imaging feasible.

## 1 Introduction

Two-photon fluorescence microscopy (TPM) is widely used to visualize the cerebrovascular structure of the rodent brain and other tissues at high spatial resolution and at extended depths.^1^^,^^2^ The imaging procedure for blood vessels involves an intravenous injection of a fluorophore that labels the blood plasma in the blood vessels. The fluorescent vascular label can then be excited via a two-photon excitation process. Common vascular labels for two- or three-photon microscopy include dextran-conjugated Texas red,^3^^,^^4^ Fluorescein isothiocyanate-conjugated dextran (FITC-dextran),^5^ and Alexa Fluor dyes.^6^^–^^8^ In the past, deep and extended two-photon imaging of the vascular network has been illustrated using these fluorescent dyes.

Although widely used vascular probes such as dextran-conjugated Texas red and Alexa Fluor dyes are effective, they are often prohibitively expensive, costing ∼$50 and $400–500 per injection in mice, respectively, depending on the variant. By contrast, indocyanine green (ICG) offers a significantly more affordable alternative at just $0.80 per injection and is already FDA-approved. Its broad clinical utility has also led to extensive research on its efficacy, safety, and applications. These factors together motivate the adoption of ICG as a vascular label for two-photon microscopy (TPM).

Despite its advantages, ICG has two key limitations as a vascular label for two-photon microscopy (TPM). First, its peak two-photon excitation wavelength overlaps with the one-photon water absorption band near 1450 nm, making conventional two-photon excitation impractical.^9^ Second, ICG is rapidly cleared from the bloodstream. Previous work by Miller et al.^10^ reported a vascular retention time of ∼20 to 25 min in mice during TPM, limiting its suitability for longer imaging sessions. In this study, we propose that encapsulating ICG within liposomal nanoparticles can enhance its circulation time and thereby enable improved two-photon fluorescence imaging.

Lipid-based nanoparticles, specifically liposomes, are one of the most heavily researched and commercialized nanoparticle systems.^11^ They have seen widespread use, approval, and licensing for everything from their earliest use to encapsulate doxorubicin for cancer therapy^12^ to their more recent use as carriers for the mRNA-based COVID-19 vaccines.^13^ Liposomal nanoparticles are inexpensive, biocompatible, biodegradable, easy to prepare, relatively stable, and highly scalable. Previous success has pushed a vast majority of lipid-based nanoparticle research to continue its focus on drug delivery, but increased circulation time and lower toxicity also suggest that liposomes may also be excellent carriers for exogenous contrast agents such as ICG.^14^

Liposomes are an ideal candidate to prolong the circulation of ICG as they do not significantly shift or dampen the absorbance or fluorescence properties of the ICG.^15^^,^^16^ In fact, several groups are currently exploring the use of liposomal ICG (L-ICG) in various forms and compositions for a variety of imaging modalities including fluorescent imaging,^17^^–^^21^ multispectral optoacoustic tomography,^15^^,^^22^ photoacoustic imaging,^23^^–^^26^ and photothermal therapy^27^^–^^29^ Applications span several organ systems including the eyes^30^ and brain^31^ as well as multiple use cases including imaging the vasculature system and monitoring tumor progression. Separately, Kraft and Ho^20^ and Gao et al.^17^ show that encapsulation within liposomes actually improves the stability and fluorescence intensity of ICG for *in vivo* applications.^17^^,^^20^ In the latter case, L-ICG shows a 38.7-fold increase in near infrared fluorescence intensity compared with free ICG likely due to the dense packing of individual ICG molecules in the liposomal shell.

In spite of these advantages, capitalizing on the retention time benefits of liposomal encapsulation for two-photon microscopy of microvasculature in the brain has not been explored. Here, we detail a procedure for the formation of ICG-encapsulated liposomes along with relevant characterization. We perform independent *in vivo* imaging of the cortical vascular network labeled with free ICG and liposomal ICG (L-ICG). Finally, we undertake a quantitative comparison between the retention of free ICG and L-ICG.

## 2 Methods

### 2.1 Synthesis of Liposomal Nanoparticles

**Fig. 1:**
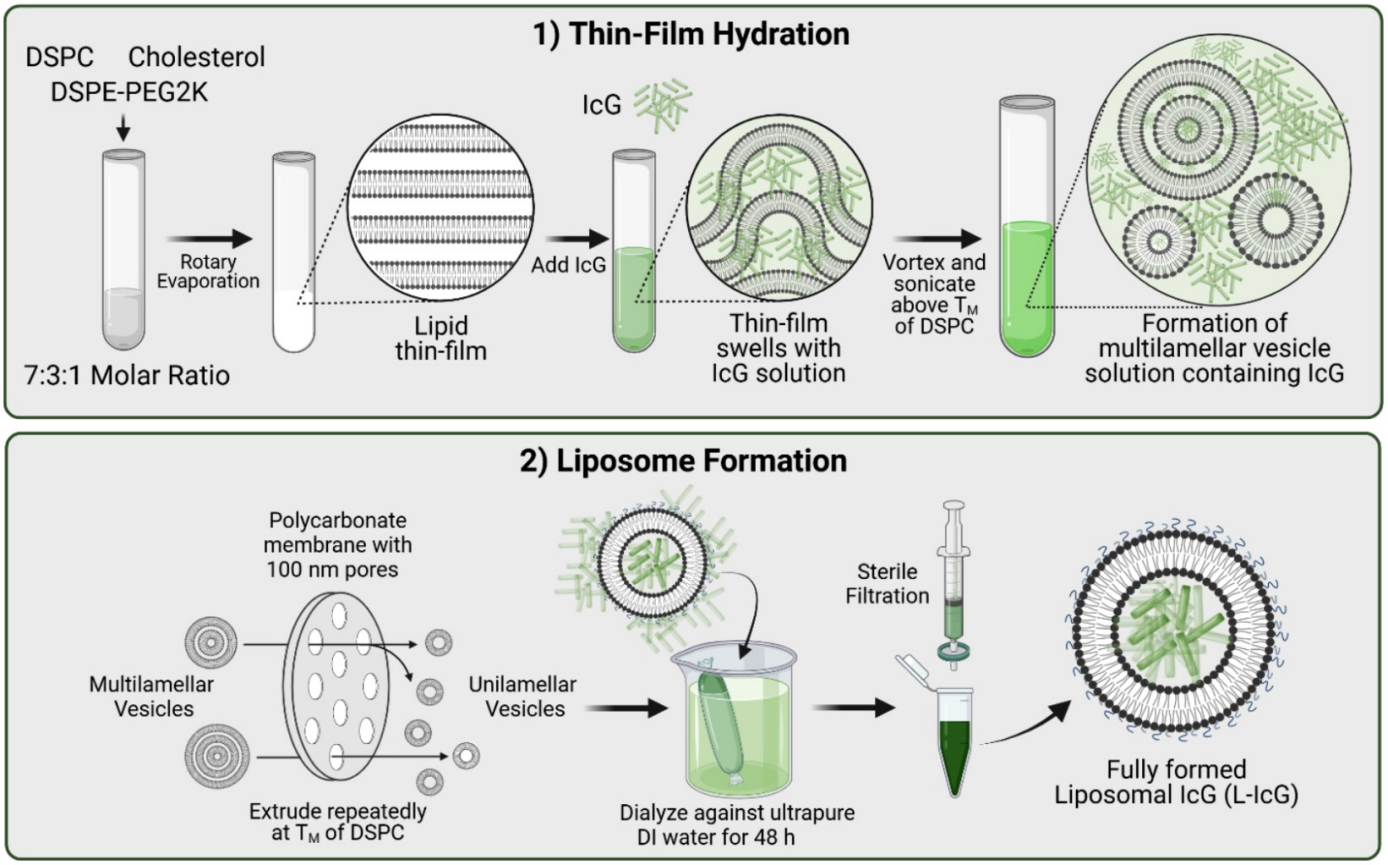
Schematic depicting the two-step process for the synthesis of liposomal ICG particles. Phase 1 encompasses thin film hydration. In this phase, lipids are added at a defined concentration, evaporated to form a thin film, and hydrated with a solution containing ICG. The resulting solution containing multilamellar vesicles with ICG moves to phase 2, where liposomes are formed. In this phase, particles are extruded down to the proper size forming liposomes, dialyzed to remove any excess ICG, and sterile filtered to remove any contaminants and larger aggregates that still remain. For *in vivo* experimentation, an additional step of centrifugation and resuspension to the desired final concentration in sterile saline or PBS is performed. Refer to the Supplementary Material for a detailed description of the synthesis process. Schematic created with BioRender.

Encapsulation of ICG within liposomes consists of three major steps: thin-film hydration, extrusion, and dialysis. Briefly, a thin film of lipids DSPC, cholesterol, and DSPE-PEG2k is formed in a defined ratio along the walls of a glass vial under vacuum. Next, the film is hydrated with a high-concentration solution of ICG and is gently heated and agitated to encourage nanoparticle formation. Once sufficiently hydrated, the resulting nanoparticle solution is extruded through 100 nm membranes to cut them down to size and increase uniformity. After extrusion, the liposomal solution is dialyzed with ultrapure water to remove any unencapsulated ICG. For *in vivo* applications, the nanoparticle solution is passed through sterile membranes and centrifuged to bring them to the desired concentration (1  mg/ml) for injection. A schematic of the entire process is shown in Fig. 1. The synthesis process is described in further detail in the Supplementary Material.

### 2.2 Characterization of Liposomal Nanoparticle

Size, zeta potential, particle concentration, encapsulation efficiency, and single-photon fluorescence intensity are the main areas of characterization. Size, zeta potential, and particle concentration are obtained through analysis with a Malvern Zetasizer and NanoSight. Encapsulation efficiency is calculated as a ratio of the initial amount of ICG used during thin-film hydration to the final amount of ICG encapsulated within the nanoparticle. Concentrations of ICG are determined after mixing in a 1:1 ratio with ethanol and comparing with a standard curve. Single-photon fluorescence intensity is measured using a Shimadzu spectrofluorophotometer. The characterization results and details are described in the Supplementary Material.

### 2.3 Ultrafast Laser Sources

The laser source setup consists of a noncollinear optical parametric amplifier (NOPA, Spectra Physics), which was pumped by a 1-MHz repetition rate laser (Spirit 1030-70, Spectra-Physics) at 35 W. The NOPA offered independent tunability of the wavelength across a wide range of wavelengths, but for the purpose of the experiments described in this work, the output wavelength was set at 1300 nm.

### 2.4 Multiphoton Microscope

**Fig. 2:**
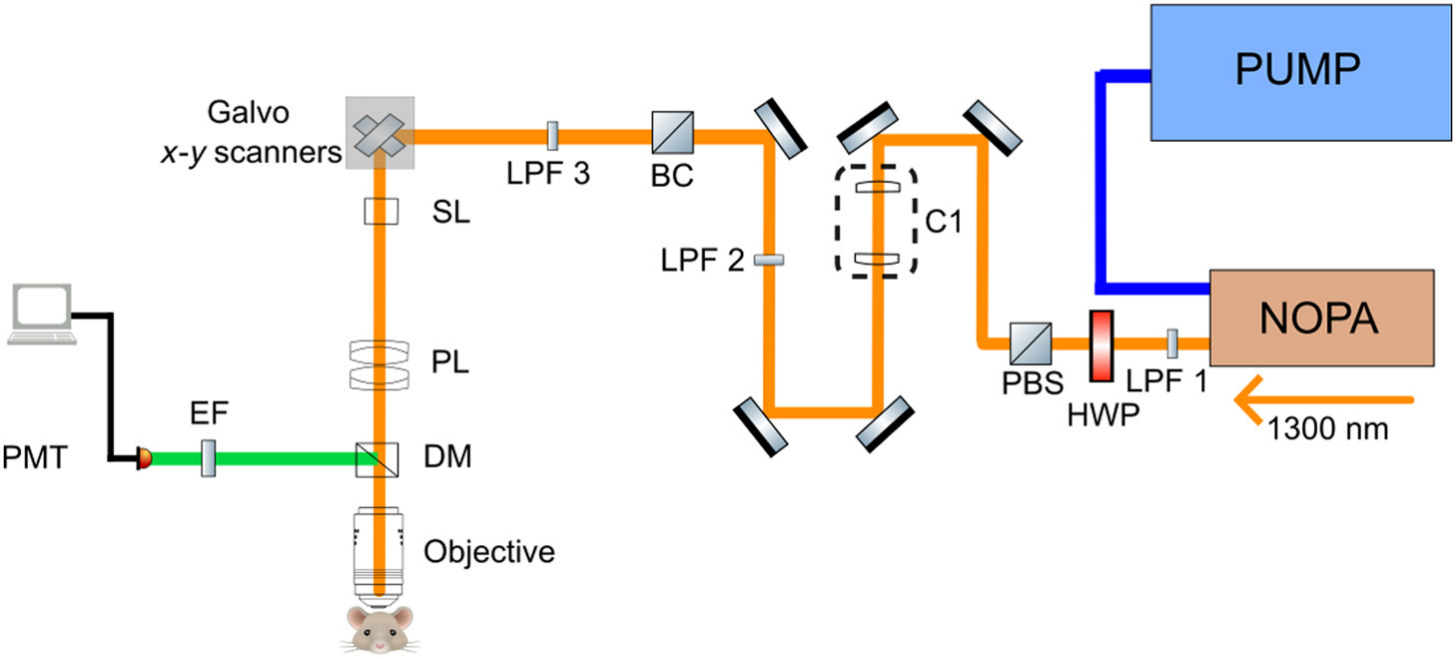
Layout of laser and imaging setup for two-photon microscopy. NOPA, noncollinear parametric amplifier; LPF, long pass filter; HWP, half-wave plate; PBS, polarizing beam splitter; C1, collimator; BC, beam combiner; SL, scan lens; TL, tube lens; DM, dichroic mirror; EF, emission filters; PMT, photomultiplier tube.

Imaging was performed using a custom home-built two-photon microscope.^3^^,^^9^ A schematic of the microscope layout is shown in Fig. 2. Longpass filters (LPF 1: FEL0850 [Thorlabs], LPF 2: FELH1150 [Thorlabs], LPF 3: FF01-937 [Thorlabs]) were used to remove any unwanted residual wavelengths in the NOPA output. A combination of a half-wave plate (HWP) and a polarizing beam splitter (PBS) is used to modulate the power of the excitation beams during the experiment. The excitation beam was raster scanned during the experimental procedure with two galvanometric scan mirrors. A scan lens (f=50  mm, SL50-3P, Thorlabs) and a Plössl tube lens were used in conjunction (f=200  mm, 2× AC508-400-C, Thorlabs) to expand the beam to fill the back aperture of a 25× objective (XLPLN25XSVMP2, 1.0 NA, Olympus). The objective focuses the excitation light onto the sample leading to emission of a fluorescence signal. The emitted photons were reflected towards the detection path by a dichroic mirror (FF980-DI01-T1, Semrock), wherein they passed through a set of emission filters (893/209, Semrock; 855/210, Semrock) before getting collected by photo-multiplier tubes (H10770PB-50, Hamamatsu). The control of the stages, scanners, and image acquisition was carried out by custom-made software in LabVIEW, and the image analysis was undertaken using Fiji.

### 2.5 Animal Protocols

The Institutional Animal Care and Use Committee at the University of Texas at Austin approved all the animal procedures. All mice were male mice and C57BL/6 strain. Isoflurane was used as our anesthetic agent (1.5% to 2%) for all surgical procedures. The cranial window site was chosen to the right of the sagittal suture between the coronal and lambdoidal sutures. A circular flap, ∼5  mm in diameter, was drilled over which the cranial window was positioned. The mouse’s temperature was maintained at 37.6°C during the craniotomy procedure. Imaging procedures were performed after allowing the mice a rest of 2 weeks. Further, all imaging procedures were undertaken with anesthetized mice.

## 3 Results

### 3.1 *In Vivo* Procedures and Signal Variation with Time

The objective of the *in vivo* studies was to compare the retention of free ICG and L-ICG from the rodent vascular network as a function of time. A total of five *in vivo* imaging procedures were performed using L-ICG, with the results from one of these (Mouse E) presented below. In addition, two *in vivo* imaging procedures were conducted using free ICG. The Supplementary Material includes L-ICG imaging results from two other mice, mouse B and mouse D. The letter designations are used solely as a naming convention; mouse A and mouse C were part of the same cohort of mice but were not part of the experiments undertaken as part of this work. In this work, the term *retention time* refers to the time after injection beyond which reasonable vascular images can not be obtained. Although this is a subjective definition, accurately quantifying retention time is not crucial; it serves merely as a metric to compare ICG retention in the body.

The L-ICG compound was prepared to a concentration of 1  mg/ml. A solution of free ICG with a similar concentration was also prepared. The blood plasma of the vascular network in the cortex was labeled through a 200-μL retro-orbital injection of either the L- or free ICG solution. Imaging was performed with an excitation wavelength of 1300 nm, which is a suitable wavelength for excitation of ICG-based compounds. The “Discussion” section provides more information on the choice of wavelength used. During imaging, the average power incident on the surface was increased with depth but did not exceed 40 mW.

After injection, the first few minutes were spent setting up the mouse under the multiphoton microscope and finding suitable regions for imaging at different depths. Imaging of the vascular network began 15 min after the injection and continued at regular 15-min intervals up to a total of 75 min. The deepest depth investigated was 600  μm for all mice that were imaged.

**Fig. 3:**
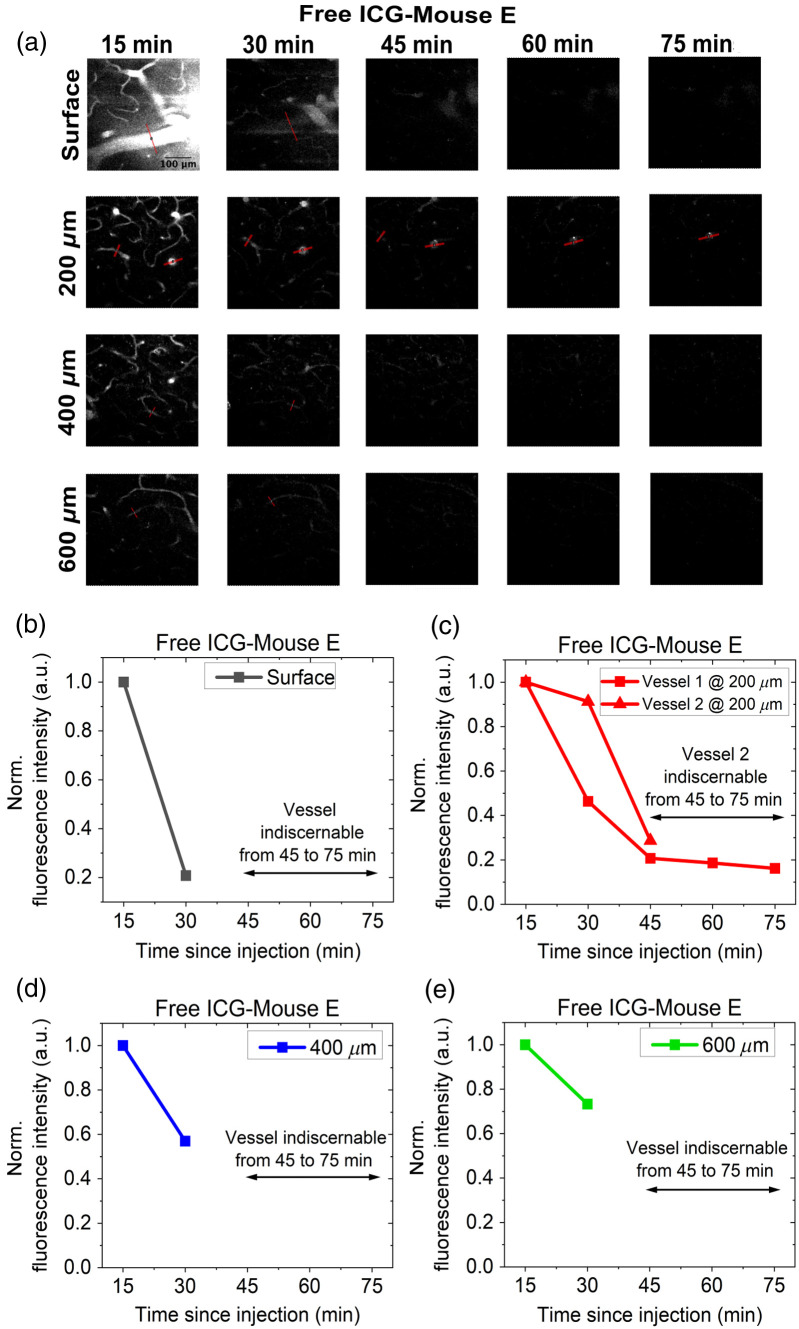
(a) *In vivo* imaging of blood vessels at different depths and time points for mouse E using free ICG. All images are displayed on the same histogram display range. The scale bar represents 100  μm. Variation of overall mean fluorescence intensity for vessels at surface (b), 200  μm (c), 400  μm (d), and 600  μm (e). The mean vessel fluorescence intensity at each time point is normalized to the value at the 15-min time point.

**Fig. 4:**
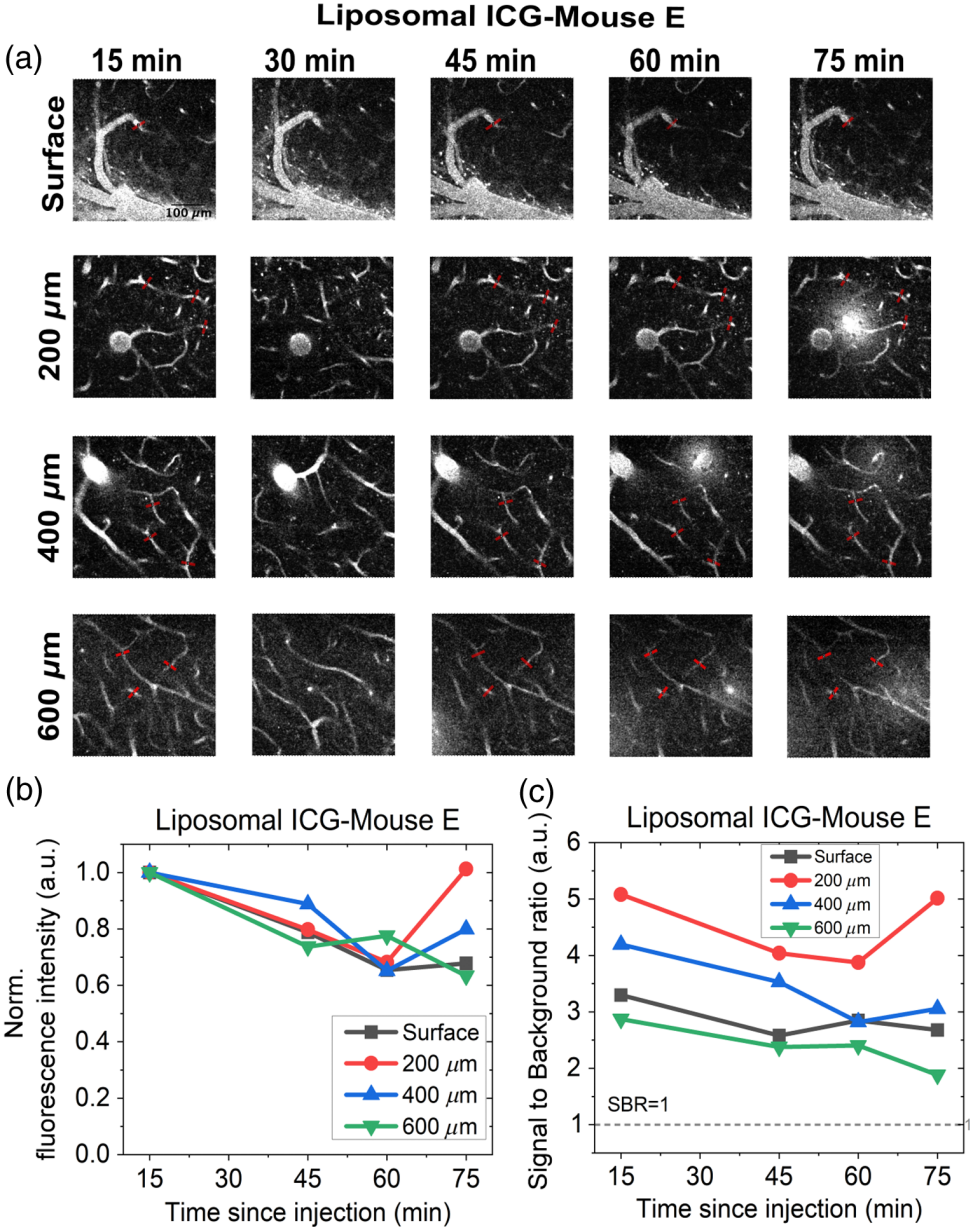
(a) *In vivo* imaging of blood vessels at different depths and time points for mouse E using L-ICG. All images are displayed on the same intensity range. The scale bar represents 100  μm. (b) Variation of overall mean fluorescence intensity for vessels at all depths. The overall mean fluorescence intensity at each time point is normalized to the value at the 15-min time point. (c) Signal-to-background ratio (SBR) for different depths and time points. The SBR for a given depth at a specific time point was calculated by averaging the SBRs of three vessels at that depth. Only 1 vessel at the surface was chosen for analysis.

Figures 3 and 4 show the imaging results and analysis from the experiments with free ICG and L-ICG, respectively. The vascular images in Fig. 3(a) are displayed using a uniform histogram scale, as are the images in Fig. 4(a). However, the histogram scales used in Figures 3(a) and 4(a) differ from each other. The retention of the dye over time can be qualitatively inspected from the images, but there is value in quantitatively analyzing the results. For the quantitative analysis, the fluorescence intensity of vessels was tracked over time. Specifically, the mean fluorescence intensity for each vessel was determined by averaging the five highest pixel intensities from the line profile of the vessel. In addition, when it was possible to track and quantify multiple vessels, two to three similarly sized vessels (depicted with red bars) were selected from each image, and their mean fluorescence intensities were averaged to obtain a value representing the overall mean fluorescence intensity of the vessels at a given depth. The overall mean fluorescence intensity at each depth was normalized relative to the value at the initial time point, i.e., 15 min. For a certain depth, this will be referred to as the normalized fluorescence intensity. When possible, the signal-to-background ratios (SBR) for the vessels were also investigated. The SBR is quantified as $(\mu_{\mathrm{sig}})/\mu_{\mathrm{bg}}$ where $\mu_{\mathrm{sig}}$ denotes the mean fluorescence intensity (as defined above) and $\mu_{\mathrm{bg}}$, mean background intensity, is found by averaging intensity values of 10 pixels on either end of the line profiles. The overall SBR (denoted by SBR in the figures) for a given depth at a specific time point was calculated by averaging the SBRs of all vessels analyzed at that depth. Note that an SBR of 1 denotes that the signal cannot be differentiated from the background.

Figure 3(a) shows the results for the *in vivo* imaging procedure with free ICG. It is observed in the images that the vessels are clearly visible up until the 15-min time point, but they are not discernible from the 30-min time point and beyond. At later time points, the vessel signal became indistinguishable from the background in the images, making it impossible to obtain any quantitative measurements of fluorescence in the vessels. Nonetheless, the signal from a single vessel was evaluated for the first two time points for the surface, 400 and 600  μm depths. At 200  μm, the signal from two vessels could be evaluated during the first two time points; however, only one vessel could be consistently tracked throughout the entire imaging session. Figure 3(b) shows the variation of normalized fluorescence intensity as a function of time. The graphs in Fig. 3(b) show a significant drop in fluorescence intensities between 15 and 30 min.

Figure 4(a) shows the images obtained from *in vivo* experiments using L-ICG. It can be seen that at the same depth, the field of view is similar at all time points. Note that the imaging plane at the 30-min time point was slightly shifted for depths of 200  μm and beyond. For this reason, the images obtained at 30 min were excluded from the data analysis shown in Figs. 4(b) and 4(c). A quantitative understanding of the retention of the dye can be obtained from Fig. 4(b). Figure 4(b) shows that for all depths the normalized fluorescence intensity decreases over the duration of the imaging experiment, but the decrease is gradual. Even at the 75-min time point, the vessels were as bright as 60% to 80% of their initial brightness. Similar results were observed in other mice imaged with L-ICG, with normalized fluorescence intensity remaining above 50% at all depths for at least 75 min after injection (Figs. S3 and S4 in the Supplementary Material). Although variability in the normalized fluorescence intensity among the mice was observed, it did not alter this overall finding. Table S1 in the Supplementary Material presents the normalized fluorescence intensity using liposomal ICG in Mice E, B, and D as a function of time across all depths.

Although Fig. 4(b) shows the overall signal trend within the vessels over time, we also examined the effect of dye clearance on the SBR. This is presented in Fig. 4(c), where the SBR is seen to gradually decrease over time.

## 4 Discussion

Fluorescence imaging with ICG offers notable advantages, including low cost, accessibility, safety, and extensive availability of research. However, two-photon imaging of blood vessels labeled with ICG is limited by its relatively short clearance time. Although a short imaging window of ∼15 to 20 min may suffice for brief experiments, it is unsuitable for longer experiments that require deeper imaging or higher frame averaging. This time window is particularly limited, as it must accommodate positioning the rodent under the microscope and identifying a suitable cortical region for imaging.

A method to extend imaging duration with ICG is by injecting a second retro-orbital dose to replenish the dye in the vessels, enabling deeper imaging. Although effective, this approach is not ideal, as re-injecting the dye once the mouse is positioned under the microscope is challenging. Furthermore, repeated injections may have undesirable effects on the rodent.

In this work, we propose liposomal encapsulation as a method to overcome the issue of rapid clearance of ICG from the rodent body. The mechanism of clearing of ICG involves binding to the plasma proteins and consequent clearing by the liver or the kidney. We propose that the binding of the ICG particles to the plasma proteins can be prevented by encapsulating ICG in liposomal nanoparticles. In this way, the retention time for ICG in the rodent vascular network can be increased. Liposomal nanoparticles have been widely researched, are easily available, and are simple to synthesize. Further, the synthesis process adds negligible costs and is easily implementable through standard processes available in any wet lab.

As shown in Fig. S1 in the Supplementary Material, encapsulating ICG in liposomal carriers does not alter its one-photon fluorescence intensity or absorbance spectra. Although a similar outcome was anticipated for two-photon excitation, we wanted to experimentally confirm this. Figure S2 in the Supplementary Material presents the normalized two-photon brightness spectra for both L-ICG and free ICG as a function of excitation wavelength (1250 to 1600 nm) across different power levels. These spectra indicate the range over which the fluorophores can be efficiently excited via two-photon excitation. The results reveal that L-ICG and free ICG exhibit similar spectral profiles, with both showing efficient excitation at 1300 nm. Based on this finding, all imaging experiments were performed using two-photon excitation at 1300 nm.

As seen in the *in vivo* imaging results with free ICG in Fig. 3(a), the vessels are clearly visible up to the 15-min time point but become indiscernible from 30 min onward. This is due to the rapid clearance of free ICG. This observation is further supported by the graphs in Fig. 3(b), which show a significant decline in fluorescence intensity between 15 and 30 min. These findings indicate that substantial clearance of ICG occurs within 15 to 20 min post-injection, consistent with previous reports by Miller et al.^10^ There is one specific vessel at 200  μm that was visible through the course of the entire imaging session. As seen in Fig. 3(b), the fluorescence intensity of this vessel decreases to 40% of its initial value at 15 min by 30 min and further declines to 20% by 45 min. The normalized fluorescence intensity then plateaus at ∼20% and remains stable until the end of the imaging session at 75 min. Although this vessel remains visible for extended durations, it represents an outlier, and any quantification of clearance from this vessel would likely be an overestimation.

In contrast to the rapid clearance observed with free ICG, the *in vivo* imaging results using L-ICG, shown in Fig. 4(a), reveal a substantially different trend. A key observation from these images is that the vessels remain clearly distinguishable from the background at all time points and imaging depths. This prolonged visibility is particularly notable given that the same laser power was used across all time points for a given depth. Interestingly, at certain time points, such as the 75-min mark at 200  μm, vessel ablation is observed, possibly due to the high peak powers used for imaging.^32^^,^^33^ Notably, the ablation appears to generate excess signal within the field of view, resulting in an increase in normalized fluorescence intensity. This effect is visible in Fig. 4(b) at the 200  μm depth.

The gradual decrease in SBR over time, as shown in Fig. 4(c), is attributed to the slow clearance of L-ICG. Despite the clearing, the SBR remains well above 1 across all depths and time points, indicating that the vessels remain clearly distinguishable from the background throughout the imaging session. In this particular experiment, the SBR for surface vessels is observed to be lower than that for deeper vessels. The following paragraph outlines our interpretation of this observation.

The SBR is influenced by two key factors: (i) the mean signal intensity from the vessels and (ii) the mean background intensity in the vicinity of the vessel. Consequently, SBR is highly sensitive to variations in the background. From the analysis, it was observed that the same vessel at different time points could exhibit significant variance in the mean background. This may result from artifacts near the vessel, causing an unexpectedly high background, or from extremely low background levels, leading to an unusually high SBR. Therefore, while generating line profiles, careful attention was paid to avoid regions on the vessels that might encounter either of these issues. It was difficult to completely avoid this, and so, the absolute values for the SBRs at different depths cannot be compared accurately. Because our focus is on assessing dye clearance, we are primarily concerned with the trend of SBR over time at various depths rather than its exact values.

A comparison of the results in Figs. 3 and 4 demonstrates that liposomal encapsulation significantly enhances the retention of ICG within the blood vessels. Whereas free ICG clears within ∼20  min, encapsulated ICG remains detectable in the vasculature for up to 75 min. The higher retention of L-ICG was observed across all five animals that were imaged as part of this work. It is also worth noting that the retention of liposomal ICG is comparable with that of other commonly used dextran-conjugated fluorescent dyes for two-photon imaging, such as Texas red^10^ and rhodamine B.^34^ Further, L-ICG likely persists beyond 75 min; however, imaging was not extended past this time point in the current study. An extended imaging window would facilitate a smoother experimental workflow and enable studies requiring longer durations, such as deep vascular imaging.

## 5 Conclusion

ICG is an FDA-approved, accessible vascular label for two-photon imaging. Its rapid clearance from the rodent body serves as one of the limiting factors for *in vivo* two-photon imaging. We propose a safe and inexpensive technique to improve the retention of ICG that involves the encapsulation of ICG particles in liposomal nanoparticles. We compare the retention up time for nonencapsulated or free ICG and L-ICG for *in vivo* imaging and note that encapsulation improves the clearance time for ICG from 20 min to at least 75 min. This work is an attempt to improve the methods for two-photon microscopy with ICG. Given the wide prevalence of ICG in clinical settings, these improved methods could be vital toward translating two-photon microscopy to the clinic.

## Supplementary Material

10.1117/1.JBO.30.9.096004.s01

## Data Availability

Raw images and data are not publicly available at this time but may be obtained from the authors upon reasonable request.

## References

[1] Helmchen F. Denk W., “Deep tissue two-photon microscopy,” Nat. Methods 2(12), 932–940 (2005).1548-7091 10.1038/nmeth818 16299478

[2] Shih A. Y. et al., “Two-photon microscopy as a tool to study blood flow and neurovascular coupling in the rodent brain,” J. Cereb. Blood Flow Metab. 32, 1277–1309 (2012). Erratum in: J Cereb Blood Flow Metab. 2013 Feb;33(2):319. Dosage error in article text. 10.1038/jcbfm.2011.196 22293983 PMC3390800

[3] Zhou A. et al., “Evaluation of resonant scanning as a high-speed imaging technique for two-photon imaging of cortical vasculature,” Biomed. Opt. Express 13, 1374–1385 (2022). BOEICL 2156-7085 10.1364/BOE.448473 35414984 PMC8973172

[4] Hontani Y. Xia F. Xu C., “Multi-color three-photon fluorescence imaging deep in mouse brain with enhanced cross section,” in Opt. InfoBase Conf. Pap., Part F181, March (2020). 10.1364/CLEO_AT.2020.JTh4A.1

[5] Levene M. J. et al., “In vivo multiphoton microscopy of deep brain tissue,” J. Neurophysiol. 91(4), 1908–1912 (2004). JONEA4 0022-3077 10.1152/jn.01007.2003 14668300

[6] Shen Z. et al., “An artery-specific fluorescent dye for studying neurovascular coupling,” Nat. Methods 9, 273–276 (2012).1548-7091 10.1038/nmeth.1857 22266543 PMC3392962

[r7] Kobat D. Horton N. G. Xu C., “In vivo two-photon microscopy to 1.6-mm depth in mouse cortex,” J. Biomed. Opt. 16, 106014 (2011). JBOPFO 1083-3668 10.1117/1.3646209 22029361

[8] Engelmann S. A. et al., “Diamond Raman laser and Yb fiber amplifier for in vivo multiphoton fluorescence microscopy,” Biomed. Opt. Express 13, 1888–1898 (2022). BOEICL 2156-7085 10.1364/BOE.448978 35519268 PMC9045921

[9] Tomar A. et al., “Non-degenerate two-photon imaging of deep rodent cortex using indocyanine green in the water absorption window,” Biomed. Opt. Express 15, 5053–5066 (2024). BOEICL 2156-7085 10.1364/BOE.520977 39296386 PMC11407249

[10] Miller D. R. et al., “In vivo multiphoton imaging of a diverse array of fluorophores to investigate deep neurovascular structure,” Biomed. Opt. Express 8(7), 3470 (2017). BOEICL 2156-7085 10.1364/BOE.8.003470 28717582 PMC5508843

[11] Navya P. N. Daima H. K., “Rational engineering of physicochemical properties of nanomaterials for biomedical applications with nanotoxicological perspectives,” Nano Converg. 3, 1 (2016). 10.1186/s40580-016-0064-z 28191411 PMC5271116

[12] Barenholz Y., “Doxil®–the first FDA-approved nano-drug: lessons learned,” J. Control. Release 160, 117–134 (2012). JCREEC 0168-3659 10.1016/j.jconrel.2012.03.020 22484195

[13] Nsairat H. et al., “Liposomes: structure, composition, types, and clinical applications,” Heliyon 8, e09394 (2022). 10.1016/j.heliyon.2022.e09394 35600452 PMC9118483

[14] Kumari A. Kumari K. Gupta S., “The effect of nanoencapsulation of icg on two-photon bioimaging,” RSC Adv. 9, 18703–18712 (2019). 10.1039/C9RA03152A 35515210 PMC9064784

[15] Song W. et al., “Comprehensive studies of pharmacokinetics and biodistribution of indocyanine green and liposomal indocyanine green by multispectral optoacoustic tomography,” RSC Adv. 5, 3807–3813 (2015). 10.1039/C4RA09735A

[16] Huang X. et al., “Ge11 peptide conjugated liposomes for EGFR-targeted and chemophotothermal combined anticancer therapy,” Bioinorg. Chem. Appl. 2021, 5534870 (2021). 10.1155/2021/5534870 33868396 PMC8035035

[17] Gao D. et al., “Low-dose NIR-II preclinical bioimaging using liposome-encapsulated cyanine dyes,” Small 19, e2206544 (2023). SMALBC 1613-6810 10.1002/smll.202206544 36710248

[r18] Zhang Y. et al., “In vivo pharmacokinetics assessment of indocyanine green-loaded nanoparticles in tumor tissue with a dynamic diffuse fluorescence tomography system,” Mol. Imaging Biol. 21, 1044–1053 (2019). 10.1007/s11307-019-01340-7 30850969

[r19] He M. et al., “Protein-enhanced NIR-IIb emission of indocyanine green for functional bioimaging,” ACS Appl. Bio Mater. 3, 9126–9134 (2020). 10.1021/acsabm.0c01384 35019590

[r20] Kraft J. C. Ho R. J., “Interactions of indocyanine green and lipid in enhancing near-infrared fluorescence properties: the basis for near-infrared imaging in vivo,” Biochemistry 53, 1275–1283 (2014). 10.1021/bi500021j 24512123 PMC3985908

[21] Mazza M. et al., “Liposome-indocyanine green nanoprobes for optical labeling and tracking of human mesenchymal stem cells post-transplantation in vivo,” Adv. Healthc. Mater. 6 (2017). 10.1002/adhm.201700374 28777501

[22] Beziere N. et al., “Dynamic imaging of pegylated indocyanine green (ICG) liposomes within the tumor microenvironment using multi-spectral optoacoustic tomography (MSOT),” Biomaterials 37, 415–424 (2015). BIMADU 0142-9612 10.1016/j.biomaterials.2014.10.014 25453969

[23] Wood C. A. et al., “Clinically translatable quantitative molecular photoacoustic imaging with liposome-encapsulated ICG J-aggregates,” Nat. Commun. 12, 5410 (2021). NCAOBW 2041-1723 10.1038/s41467-021-25452-3 34518530 PMC8438038

[r24] Wang R. et al., “Antibody-conjugated liposomes loaded with indocyanine green for oral targeted photoacoustic imaging-guided sonodynamic therapy of Helicobacter pylori infection,” Acta Biomater. 143, 418–427 (2022). 10.1016/j.actbio.2022.02.031 35219867

[r25] Wu C. et al., “Indocyanine green-loaded liposomes-assisted photoacoustic computed tomography for evaluating in vivo tumor penetration of liposomal nanocarriers,” Micromachines 15, 90 (2023). 10.3390/mi15010090 38258209 PMC10820658

[26] Humbert J. et al., “Comparison of photoacoustic and fluorescence tomography for the in vivo imaging of ICG-labelled liposomes in the medullary cavity in mice,” Photoacoustics 20, 100210 (2020). 10.1016/j.pacs.2020.100210 33101928 PMC7569329

[27] Liao W.-T. et al., “Indocyanine-green-loaded liposomes for photodynamic and photothermal therapies: inducing apoptosis and ferroptosis in cancer cells with implications beyond oral cancer,” Pharmaceutics 16, 224 (2024). 10.3390/pharmaceutics16020224 38399278 PMC10891763

[r28] Stern N. B. Shrestha B. Porter T., “A facile approach to producing liposomal J-aggregates of indocyanine green with diagnostic and therapeutic potential,” Adv. Therapeutics 7, 2400042 (2024). 10.1002/adtp.202400042 PMC11308451 39132131

[29] Yoon H. J. et al., “Liposomal indocyanine green for enhanced photothermal therapy,” ACS Appl. Mater. Interfaces 9(7), 5683–5691 (2017). AAMICK 1944-8244 10.1021/acsami.6b16801 28152314

[30] Hua J. et al., “In vivo imaging of choroidal angiogenesis using fluorescence-labeled cationic liposomes,” Mol. Vision 18, 1045–1054 (2012). PMC3351413 22605917

[31] Portnoy E. et al., “Indocyanine green liposomes for diagnosis and therapeutic monitoring of cerebral malaria,” Theranostics 6(2), 167–176 (2016). 10.7150/thno.13653 26877776 PMC4729766

[32] Nishimura N. et al., “Targeted insult to subsurface cortical blood vessels using ultrashort laser pulses: three models of stroke,” Nat. Methods 3, 99–108 (2006).1548-7091 10.1038/nmeth844 16432519

[33] Vogel A. Venugopalan V., “Mechanisms of pulsed laser ablation of biological tissues,” Chem. Rev. 103(2), 577–644 (2003). CHREAY 0009-2665 10.1021/cr010379n 12580643

[34] Maurin M. et al., “Deep in vivo two-photon imaging of blood vessels with a new dye encapsulated in pluronic nanomicelles,” J. Biomed. Opt. 16(3), 036001 (2011). JBOPFO 1083-3668 10.1117/1.3548879 21456865 PMC4020796

